# Demonstrating vaccine effectiveness during a waning epidemic: A WHO/NIH meeting report on approaches to development and licensure of Zika vaccine candidates

**DOI:** 10.1016/j.vaccine.2018.12.040

**Published:** 2019-02-04

**Authors:** Kirsten S. Vannice, M. Cristina Cassetti, Robert W. Eisinger, Joachim Hombach, Ivana Knezevic, Hilary D. Marston, Annelies Wilder-Smith, Marco Cavaleri, Philip R. Krause

**Affiliations:** a Department of Immunization, Vaccines and Biologicals, World Health Organization, Geneva, Switzerland; b National Institute of Allergy and Infectious Diseases, National Institutes of Health, Bethesda, MD, USA; c Department of Essential Medicines and Health Products, World Health Organization, Geneva, Switzerland; d European Medicines Agency, London, UK; e Center for Biologics Evaluation and Research, Food and Drug Administration, Rockville, MD, USA

**Keywords:** Zika vaccine, Vaccine licensure, Immunogenicity, Human challenge trials

## Abstract

Since its peak in early 2016, the incidence of Zika virus (ZIKV) cases has declined to such low levels that Phase 3 field efficacy trials may be infeasible. While great progress was made to rapidly advance several vaccine candidates into Phase 1 and 2 clinical trials, in the absence of sustained viral transmission it may be difficult to evaluate the effectiveness of ZIKV vaccine candidates by conducting traditional clinical disease endpoint efficacy studies. However, ZIKV is still circulating at low levels in some areas and is likely to re-emerge in naïve populations or in sites of prior epidemics once population immunity wanes. Therefore, the public health need for a ZIKV vaccine remains. To facilitate continued ZIKV vaccine development efforts, the World Health Organization's Initiative for Vaccine Research and the National Institutes of Health’s National Institute of Allergy and Infectious Diseases co-hosted a meeting of experts in March 2018 to identify strategies to demonstrate vaccine effectiveness in view of waning ZIKV disease incidence. This paper outlines points for consideration for developers, regulators, and other stakeholders working towards a licensed ZIKV vaccine. These deliberations may also be applicable to development of vaccines for other emerging infections where the size, unpredictability, and ephemeral nature of outbreaks makes clinical disease endpoint efficacy trials to demonstrate vaccine effectiveness infeasible.

## 1 Introduction

The World Health Organization (WHO) declared the cluster of congenital microcephaly cases and other neurological disorders reported in Brazil as a result of Zika virus (ZIKV) infection of pregnant mothers and their infants to be a Public Health Emergency of International Concern (PHEIC) in February 2016. Just one month later, external experts to WHO advised prioritizing the development of a ZIKV vaccine, acknowledging that such development of medical countermeasures remains imperative for a potential further future outbreak [1]. The R&D communities responded rapidly, with more than 40 vaccine candidates being initially evaluated in pre-clinical studies and many progressing in further development. Of these, several have advanced beyond pre-clinical studies in animals and entered Phase 1 clinical trials [2], [3], with two candidates having entered Phase 2 clinical trials [4], [5]. Multiple vaccine platforms have shown robust protection against ZIKV challenge in mice and non-human primate (NHP) models. With a strong clinical pipeline and promising results from animal studies, vaccine-mediated prevention of ZIKV disease appears feasible.

**Fig. 1:**
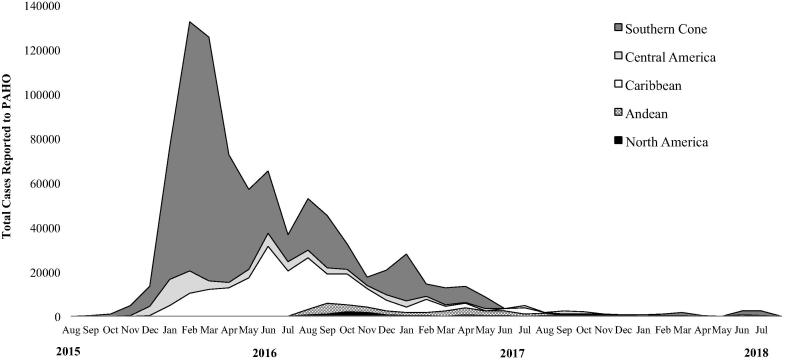
Monthly Zika illness case counts reported to the Pan American Health Organization (PAHO) during August 2015 to July 2018. Data courtesy of PAHO.

While the peak of the epidemic occurred in the first quarter of 2016 when efforts to develop ZIKV vaccines were ramped up, the number of cases in South America, the Caribbean, and Central America dropped over the course of 2016, with very low transmission remaining in 2018 (Fig. 1). Most recently, just 2,550 cases of Zika illness were reported to the Pan American Health Organization (PAHO) in July 2018, compared to a peak of over 132,000 in February 2016. These data are limited by incomplete country reporting and incomplete laboratory confirmation. As a result of the declining burden, in November 2016 WHO transitioned from the PHEIC to a sustained program of activities around Zika [6].

Despite the decline in cases, the development and licensure of a ZIKV vaccine remains a critical global public health need. The U.S. Centers for Disease Control and Prevention recently estimated approximately one in seven infants born to mothers with laboratory evidence of ZIKV infection in pregnancy were diagnosed with a Zika-associated birth defect, a neurodevelopmental abnormality possibly associated with *in utero* ZIKV infection, or both [7]. Zika continues to be on the 2018 list of WHO R&D Blueprint priority diseases [8], and WHO recently published a ZIKV vaccine roadmap with a vision for safe, effective and affordable ZIKV vaccines to prevent congenital Zika syndrome (CZS) and other serious ZIKV-associated clinical complications [3]. Low-level ZIKV transmission remains in parts of Latin America. Even in the hardest hit areas, over time herd immunity will wane thereby increasing the risk of a future outbreak upon re-introduction of ZIKV. Further global spread of Zika is likely given the increasing travel patterns [9], [10], [11]. The epidemiology of ZIKV in Asia and Africa remains poorly understood, where 2.6 billion people are estimated to live in areas at risk for ZIKV [12].

Pre-licensure clinical evaluation of vaccines typically follows a stepwise progression starting with early-stage clinical trials (e.g., Phase 1 and Phase 2) to obtain preliminary safety and immunogenicity data and information on dose and regimen of the vaccine candidate. One or more pivotal Phase 3 trials is normally used to demonstrate safety and efficacy [13]; pre-licensure clinical trials are typically randomized and controlled and are conducted in areas with sufficient disease transmission to estimate vaccine efficacy against a clinical disease endpoint [14]. With low incidence of ZIKV transmission and the unpredictable nature of future outbreaks and consequent difficulty with future field trials, opportunities to conduct randomized-controlled clinical disease endpoint efficacy trials of ZIKV vaccine candidates may be past. This presents challenges for evaluation of efficacy of ZIKV vaccine candidates. In June 2017, WHO under its R&D Blueprint hosted an expert consultation on efficacy trials of ZIKV vaccines, during which endpoints, trial design, site selection for ZIKV vaccine efficacy trials were discussed and recommendations made [15]. Since then, opportunities to conduct efficacy trials have further diminished.

To continue to facilitate ZIKV vaccine development efforts, the WHO Initiative for Vaccine Research and the National Institutes of Health (NIH), National Institute of Allergy and Infectious Diseases (NIAID) co-hosted a meeting in March 2018 to discuss strategies to demonstrate effectiveness of ZIKV vaccine candidates in the face of waning and unpredictable disease burden. While recognizing the importance of safety considerations, discussions at the meeting were limited to approaches that could be used to demonstrate ZIKV vaccine effectiveness outside of traditional field efficacy trials. WHO and NIH share the goal of bringing one or more vaccine candidates through late-stage clinical development and licensure so that a safe and effective product is ready for use when needed. This paper outlines points for consideration for developers, regulators, and other stakeholders working towards a licensed ZIKV vaccine (Box 1).

## 2 Challenges in ZIKV vaccine development

There are several challenges to ZIKV vaccine development that influence evaluation strategies for ZIKV vaccines. These include the need for well-characterized animal models relevant to human disease; the potential role of pre-existing flavivirus immunity impacting vaccine safety, immunogenicity, and/or clinical efficacy; incomplete understanding of immune responses that would be necessary to prevent fetal infection; the theoretical risk that vaccine immune responses could induce Guillain–Barré syndrome (GBS); and challenges associated with vaccination of pregnant women, potentially with a live vaccine or technologies based on platforms not previously utilized in licensed human vaccines. Current evidence suggests that even asymptomatic maternal infections with presumably low levels of viremia could result in CZS [16], thereby setting the bar high with the potential need for sterilizing immunity and robust T cell response to avert transplacental transmission of ZIKV during pregnancy [17].

The ZIKV vaccine landscape has included more than 40 candidates. Among them, nine are in early clinical development using proven technologies like attenuation and inactivation, as well as newer platforms for which no human vaccine has yet been licensed, such as DNA and modified mRNA [2]. The most advanced candidate, a DNA vaccine developed by NIAID, is currently under evaluation in a Phase 2b clinical study in multiple sites and thus may still be able to demonstrate clinical efficacy [4]. The trial is designed to measure efficacy against ZIKV disease, with efficacy against infection (symptomatic or asymptomatic) as a secondary endpoint. If the vaccine candidate is efficacious, it is hoped that immunological markers that predict protection [18] could be identified, which may also be useful in evaluation of other candidates.

## 3 Approaches to establish effectiveness in the absence of clinical disease endpoint efficacy studies

Phase 1 and Phase 2 clinical studies will be needed to establish initial safety and immunogenicity in a sufficient number of study participants before proceeding with the demonstration of vaccine efficacy in support of licensure. In the case of vaccine candidates for which traditional Phase 3 efficacy trials cannot be conducted pre-licensure, alternative approaches need to be explored. It is considered likely that a data package that supports licensure will include immunogenicity in animals and humans as well as animal challenge studies. Developers are encouraged to meet early with regulators to discuss the proposed data package and appropriate regulatory pathways.

The approach to demonstrating vaccine efficacy differs depending on the regulatory pathway to licensure. In addition, available regulatory pathways to licensure vary by country and regulatory authority. For example, in the United States, a ZIKV vaccine could be licensed by the US Food and Drug Administration (FDA) by one of three available pathways: traditional approval, accelerated approval, or the animal rule. A description of these pathways with considerations specific to Zika has been published [19].

Similarly, the European Medicines Agency (EMA) has regulatory tools and options to allow the licensure of a vaccine based on non-traditional approaches when adequately justified. Approval under exceptional circumstances and conditional marketing authorization are among the regulatory pathways that could accommodate for such scenarios. These pathways allow for flexibility in terms of the overall evidence that would be required to support the efficacy of the vaccine candidate and might include efficacy from human challenge models or extrapolation of protection to humans from adequate animal challenge models in which animals from a relevant animal species recapitulating key aspects of human disease are protected after passive transfer of human antibodies and/or after vaccination.

Regulatory pathways are available across national regulatory agencies to advance vaccine candidates without the need for a traditional clinical disease endpoint efficacy trial to demonstrate vaccine efficacy and without the existence of a scientifically well-established marker that predicts protection. In general, these pathways have in common initial licensure of a vaccine based on data that include a non-clinical endpoint in human studies and a requirement for post-licensure studies to further define or confirm benefit. For example, the US FDA Accelerated Approval regulation permits use of a surrogate marker (e.g., an immune response reasonably likely to predict the benefit of the product) or an intermediate clinical endpoint to demonstrate vaccine efficacy in support of licensure. For vaccines, this surrogate marker is usually an immunological endpoint measured in an *in vitro* assay. Assays that might be able to predict a reasonable likelihood of clinical benefit could be based on ZIKV challenge studies in mice that received passively transferred human immune serum (harvested post-vaccination). Immune markers associated with protection in animal studies and/or field efficacy studies (should incidence permit) could also be considered. The extent to which an immune marker associated with protection for a given vaccine candidate could predict efficacy of other vaccine candidates, particularly those employing differing vaccine platforms, is unknown at present. For some vaccines where the mechanism of protection is well understood, the immune marker that predicts protection is platform-independent (e.g., the protective immune response for Japanese encephalitis vaccines is relevant for both the live attenuated, live recombinant (chimeric), and inactivated vaccines) [18]; however, it is possible that it could be vaccine specific, depending on characteristics such as candidate antigen and method of delivery [19].

There are also different options for an appropriate clinical marker or intermediate endpoint that could be used as an endpoint in animal challenge studies and extrapolated to humans. One endpoint to be considered is protection against ZIKV viremia post-challenge. However, prevention of *in utero* infection in an animal pregnancy model (or in a combination of models) or demonstration of sterilizing immunity (defined by the lack of a serologic immune response, e.g., <4-fold increase in neutralizing antibody titers post-challenge) may also be useful endpoints, as they indicate more direct benefit even though they may be more difficult to attain. Feasibility of individual endpoints may also depend on the timing of challenge relative to vaccination: sterilizing immunity may be achievable four weeks after vaccination but may be more difficult to attain 12 months after vaccination.

## 4 Immune markers reasonably likely to predict clinical benefit

From a regulatory perspective, it will be useful to identify an immune marker that is reasonably likely to predict clinical benefit [18]. Such markers based on neutralizing antibodies (as measured by the Plaque Reduction Neutralization Test, or PRNT) have been defined for several flavivirus vaccines: titers of 10 are accepted as protective for Japanese encephalitis and Tick-borne encephalitis vaccines, while a titer as low as 5 has been proposed for Yellow fever vaccine [18]. For dengue, no neutralizing antibody titer has been identified, although in large Phase 3 clinical trials higher neutralizing antibody titers were associated with reduced likelihood of disease [20]. Thus, neutralizing antibody has potential as an immune endpoint to predict benefit for ZIKV vaccines.

However, while promising data exist, there are information gaps regarding potential use of neutralizing antibodies to predict clinical benefit of ZIKV vaccines. While relatively low titers are accepted as predictive of protection for vaccines against several other flaviviruses, the titer needed to protect against ZIKV may be different from other flaviviruses. Furthermore, it is unknown whether the titer required to protect against clinical disease in the vaccinee is the same as that required to protect against fetal infection. Preliminary data from NIH found that *in vitro* neutralizing antibodies of similar titers induced by two related DNA vaccine candidates were associated with different levels of protection against viremia in animal challenge studies [unpublished data]. This finding suggests that other characteristics of the neutralizing antibody (besides the titer) may need to be identified in order to predict protection across vaccine candidates. Systems vaccinology approaches have been successful in characterizing complex immune functions associated with vaccine protection for other viruses, e.g., HIV, and may have applicability to ZIKV [21].

Finally, concerns have been raised about using neutralization assays to measure ZIKV vaccine immunogenicity in individuals who have been previously exposed to other flaviviruses, either through natural infection or vaccination, due to potential cross-reactivity [22]. However, in recent longitudinal studies, patterns of antibody cross-neutralization in primary and secondary DENV infections suggest that ZIKV lies outside the DENV serocomplex [23]. Neutralizing antibody titers to ZIKV were markedly lower than to the infecting DENV and heterologous DENV serotypes. Cross-neutralization was greatest in early convalescence, then ZIKV neutralization titers decreased, remaining at low levels over time. Neutralizing antibody titers can therefore distinguish ZIKV from DENV infections during late convalescence when all viruses are analyzed simultaneously.

The assay used to measure neutralizing antibody will affect the reported numerical titer, and careful interpretation in view of the cell type used for assay, virus strain, and assay type is needed. Primary assays being used for ZIKV vaccine candidates include the traditional PRNT, microneutralization test (MN), and reporter virus particle assays. These three assays differ with respect to how viral infection is enumerated in the presence or absence of antibody, adaptability to multiple cellular substrates, and requirement for viral replication. Standardized, validated neutralization assays could help facilitate vaccine development. Several efforts are underway to support assay validation.

The National Institute for Biological Standards and Control (NIBSC) is currently in the process of validating a serology standard and a PCR standard that will be reviewed by the WHO Expert Committee on Biologicals Standardization (ECBS). WHO international standards and reference materials for designating the activity of vaccines and other biologicals can serve as a basis for evaluation of the quality, safety and efficacy of these products for the purpose of licensure as well as post-licensure. These standards can support WHO prequalification and they also allow comparability of data worldwide [24]. They are developed through the international collaborative laboratory studies and serve as the primary calibrants against which national measurement standards or working standards are benchmarked. In October 2018 the ECBS reviewed the outcomes of the collaborative study conducted by the NIBSC and adopted the 1st International Standard as Anti-Asian lineage ZIKV antibody (human) with the assigned unitage of 250 International Units per ampoule [25]. This standard could be utilized for diagnosis, vaccine evaluation and sero-surveillance.

NIH, through its contracting partners, is working to produce and characterize master and working ZIKV stocks, generate critical reagents, and develop and standardize assays. Assay development and optimization, and ultimately qualification, is underway or planned for the plaque assay, RT-qPCR, Reporter viral particles-flow neutralization (RVP-FN) assay, MN assay, and PRNT. Standardized protocols and qualified assays for human clinical trials, bridged to use in NHP challenge studies, will be made available to developers.

## 5 Results from animal challenge studies

Animal models are an important element supporting licensure of vaccines in case human efficacy studies are not feasible. Mouse challenge models to evaluate efficacy can be useful tools for comparing different candidates and to study vaccine modes of action. NHP challenge models may more closely predict human outcomes and are suitable to explore efficacy against various disease endpoints, potentially including protection against CZS, transplacental infection, GBS, and viral persistence. Passive transfer studies in which human immune sera (generated in response to infection or vaccination) is transferred into animals, which are subsequently challenged with ZIKV, may be the closest currently feasible approximation in animal studies to demonstrate the protective value of human antibodies and to infer clinical benefit in humans.

Several animal models for ZIKV infection and disease are currently being used. Murine models include immunocompetent adult, immunocompromised adult, and immunocompetent neonatal mice. NHP challenge models have been developed using rhesus macaques, cynomolgus macaques, and pigtail macaques. Guinea pig [26] and chicken embryo models [27] are also under development. Small and large animal models that could potentially be used to study vaccine effects on outcomes of ZIKV infection in pregnancy are also becoming available (e.g., [28], [29], [30]).

Vaccine data from animal challenge studies have been promising. The endpoint in most animal vaccine challenge studies is protection against viremia, measured as a lack of anamnestic response post challenge and/or lack of viremia post-challenge. Several vaccine candidates have demonstrated apparent sterilizing immunity when challenged one month following vaccination. Candidates have been successfully evaluated in NHPs with challenge one year post-vaccination [31]. In several studies with mice and NHPs, a neutralizing antibody titer of approximately 100 as measured by the MN assay was associated with protection against viremia with ZIKV challenge, while neutralizing titers lower than this were typically not protective (e.g., [32], [33], [34], [35]). This same titer was also effective in passive transfer of human immune sera obtained from study participants in Phase 1 vaccine clinical trials into mice prior to challenge [34]. Additionally, depleting CD4 + and CD8 + T lymphocytes cells did not affect vaccine protection in these mouse studies [36]. Thus, passive protection studies in mouse and NHP models support strong consideration of neutralizing antibody as a measure of immune responses likely associated with protection [32], [34]. Challenge models to test vaccine candidates for their ability to impact pregnancy outcomes may identify titer levels sufficient to protect against CZS. Preliminary findings from challenge studies in vaccinated mice indicate markedly diminished levels of ZIKV RNA in maternal, placental, and fetal tissues, which resulted in protection against placental damage and fetal demise [37]. While these findings should always be contextualized in view of potential interspecies differences, they support the proof-of-concept that protection against CZS is possible.

## 6 Controlled human infection model for Zika

Controlled Human Infection Model (CHIM) studies have been developed for several diseases, including influenza, malaria, cholera, and dengue [38]. The US FDA recently licensed a cholera vaccine on the basis of efficacy from CHIM and supportive safety and immunogenicity trials [39]. Thus, CHIM could be considered to support vaccine licensure, particularly in situations when randomized controlled field studies with a clinical disease endpoint are infeasible. WHO has summarized regulatory considerations for human challenge trials conducted as part of vaccine development [40].

CHIMs have been utilized to enhance vaccine development in several ways: (1) to assist with down-selecting vaccine candidates; (2) to study disease pathogenesis; (3) to directly evaluate vaccine efficacy and duration of protection, and (4) to identify immune markers that can predict protection. Based on these considerations, the role of a ZIKV CHIM for vaccine development has been discussed [26].

A December 2016 NIAID and the Walter Reed Army Institute of Research (WRAIR)-cosponsored expert consultation discussed conditions under which use of a ZIKV CHIM could be ethically justified. Participants raised concern that a CHIM may not be justified if traditional field efficacy trials were considered feasible, and that a ZIKV CHIM study may present risks to non-participants (third parties) if the challenge strain spread to individuals not enrolled in the study [41]. The latter could be mitigated through careful study design if the length of time of ZIKV transmission after infection was better characterized; and at the time of the report, incidence of ZIKV infection was felt to be high enough that field efficacy trials appeared feasible. Therefore, members of the expert committee felt that the social value of CHIM, when combined with other uncertainties, did not justify the potential risks of performing the studies at that time.

Fifteen months later, the calculus has changed. Traditional efficacy trials may be very difficult, if not infeasible, to conduct and the ability to directly demonstrate efficacy in a CHIM could provide the data needed for licensure. Data suggest that from ZIKV-infected individuals with low levels of viremia, ZIKV is only transmissible for 30 days [42]. Several strategies have been proposed that could greatly mitigate the risk to bystanders that are not participating in the study (such as sexual partners or fetuses), such as only enrolling females not of child-bearing age or mandating use of highly effective contraception [43]. If certain populations were not included in CHIM, immunobridging with an accepted immune marker could support potential use of vaccine in these populations. The data package and CHIM results, along with supportive safety and immunogenicity studies would determine the indication, which is generally based on the vaccine tested and what was demonstrated.

Thus, it is perceived that the situation with respect to consideration of ZIKV CHIM has changed substantially since the time of the ethics consultation in 2016, with greater appreciation for societal value and also better or improved understanding of how risks might be mitigated. Meeting participants, including representatives of regulatory agencies US FDA and EMA, agreed that CHIM may represent a useful tool for evaluating ZIKV vaccine candidates. Scientific issues for CHIMs, including the challenge strain, route of administration, dose, and timing of challenge post-vaccination will need to be addressed before CHIMs are used to evaluate vaccine efficacy.

## 7 Post-licensure studies

Regardless of the regulatory pathway used to demonstrate vaccine effectiveness for the purposes of regulatory approval, post-marketing studies will be needed for approved ZIKV vaccines. Depending on the licensure pathway, post-licensure studies may be required as confirmatory effectiveness and expanded safety studies, but even if not required by applicable regulation, it will be important to assess vaccine impact on the serious but rare sequelae of ZIKV infection.

In addition to virologically-confirmed Zika disease, likely effectiveness outcomes of interest for post-licensure studies include vaccine effectiveness against CZS and GBS, which are too rare in occurrence to serve as clinical endpoints in pre-licensure trials. Such studies are likely to be challenging in the context of a ZIKV outbreak in affected areas. The majority of ZIKV-associated CZS occurred in areas of Brazil with limited surveillance and research infrastructure. The ability to do post-licensure studies also requires vaccine rollout on a large basis, as the utility of these studies in gaining additional information on the vaccine may be limited if small select populations are vaccinated. In order to be successful, it is critical to know the vaccination status of individuals, which (depending on study design) may require a vaccine registry, and for CZS it will be important to be able to link fetal outcomes with maternal immunization status. Such infrastructure is lacking in many parts of the world, especially for capturing adult vaccinations.

Advanced preparation is therefore critical to assure the success of complex post-licensure studies. In Latin America, local ZIKV epidemics lasted at times only a few months, which is too short a time to set up a vaccination program and post-licensure study once an outbreak has emerged. Protocols should be prepared, and regulatory and ethics approvals received, in advance.

Several post-licensure study designs could be considered, including cohort and test-negative case-control designs. Because CZS presents with a wide range of clinical manifestations, intermediate markers of CZS that are routinely collected for all neonates, such as head circumference, birth weight, and gestational age, will likely need to be used as part of a case definition of CZS. Heel blood samples that are collected for metabolic screening could potentially be used to test for ZIKV antibodies. Ongoing projects, such as the WHO individual participant data-meta-analysis (IPD-MA) that will combine de-identified data on individual pregnant women from different cohorts in Latin America, will help to identify the key predictors of CZS that could be used as a pragmatic endpoint in a vaccine effectiveness post-licensure study [44]. A substantial investment in post-licensure studies will be needed.

## 8 Conclusions

Tremendous progress has been made in ZIKV vaccine development since the WHO declaration of PHEIC in February 2016. Data available from pre-clinical animal studies to date show that vaccine candidates are capable of protecting animals from subsequent challenge with ZIKV. There are several regulatory pathways for ZIKV vaccine candidate licensure if field clinical disease endpoint efficacy studies are no longer feasible in the context of a waning epidemic. Several regulatory authorities have the ability to approve vaccines when there is a reasonable likelihood of clinical benefit, allowing considerable regulatory flexibility. Based on experiences with other flavivirus vaccines, identifying immune markers associated with protection are likely feasible, but post-licensure studies would be needed to confirm clinical benefit. CHIM studies may now present the most direct opportunity to demonstrate ZIKV vaccine candidate efficacy. Manufacturers are encouraged to meet with regulators as early as possible to discuss options for regulatory pathways and post-licensure studies. While data packages without field efficacy trials may be sufficient for initial licensure, policy makers will also need data to inform decision-making for vaccine introduction. Post-licensure data might be required by regulators and would be relevant to inform and expedite public health decisions on vaccine use. Public health research agendas are critical to support aspects of vaccine development such as clinical trial site selection, and aspects following licensure, such as post-licensure studies and ZIKV transmission globally.

Box 1 **Points for consideration to advance ZIKV vaccine candidates during a waning epidemic.**

• Heterogeneity of incidence of ZIKV infection in time and space is one of the key challenges for ZIKV vaccine clinical trial design.
• Neutralizing antibodies are likely to be an important immune marker of protection; however, a broader assessment of the immune response is encouraged.
• Availability of WHO standards, standardization and validation of assays are critical for appropriate assessment of the immune response to Zika vaccine as well as for the interpretation of the results.
• Passive transfer studies in animals demonstrating protection against disease, infection, and CZS with human sera may provide a useful approximation of clinical benefit in humans in the absence of traditional efficacy trials.
• Traditional clinical disease endpoint efficacy studies may be challenging or even infeasible given the current epidemiology of ZIKV; thus, the societal benefit for CHIM is evident. With proper communications plans, risk mitigation in place, and ethical review, ZIKV CHIM could be valuable for moving forward with ZIKV vaccine development and licensure.
• Post-licensure studies will be required for all approved ZIKV vaccines: the specific studies will depend on the regulatory pathway, the indication, and vaccine characteristics.
• Many data gaps remain, and further epidemiologic research is critical to inform optimal vaccine development and use.

## Declarations

JH and IK are staff of WHO. KV and AWS served as consultants to WHO. MC is an employee of EMA. HM, CC, and RE are staff of NIH/NIAID. PRK is a staff member of US FDA.

The views expressed in this article are the personal views of the authors and may not be understood or quoted as being made on behalf of or reflecting the position of the agencies or organizations with which the authors are affiliated.
